# An Exploratory Analysis of Postural Control in People with Type 2 Diabetes Mellitus Using a Smartphone IMU Sensor

**DOI:** 10.3390/s26092899

**Published:** 2026-05-06

**Authors:** Trine Rolighed Thomsen, Sophia Pölhöšová, Asger Ahlmann Bech, Aksayan Arunanthy Mahalingasivam, Nicklas Højgaard-Hessellund Rasmussen, Anderson Souza Oliveira

**Affiliations:** 1 Department of Chemistry and Bioscience, Aalborg University, DK-9220 Aalborg, Denmark; trt@bio.aau.dk (T.R.T.); polhossophia@gmail.com (S.P.); 2 Danish Technological Institute, DK-8000 Aarhus, Denmark; 3 Steno Diabetes Center North, DK-9220 Aalborg, Denmark; asger.bech@rn.dk (A.A.B.); aksayan.mahalingasivam@rn.dk (A.A.M.); nicklas.rasmussen@rn.dk (N.H.-H.R.); 4 Department of Clinical Medicine, Aalborg University Hospital, DK-9220 Aalborg, Denmark; 5 Department of Materials and Production, Aalborg University, Fibigerstræde 16, DK-9220 Aalborg, Denmark

**Keywords:** diabetes, postural control, fall risk, wearables, smartphone, IMU

## Abstract

**Highlights:**

**What are the main findings?**

- We proposed a posturography test protocol using only the IMU embedded on a smartphone fixed at the low back area. The smartphone-based postural analysis yields similar results compared to traditional center-of-pressure analysis.
- The smartphone-based postural analysis was able to identify specific deficits in patients with type 2 diabetes that were not present in healthy individuals.

**What are the implications of the main findings?**

- Temporal analysis of IMU data from regular smartphones is suitable for relevant postural analysis in healthy people and patients with type 2 diabetes.
- The proposed analysis has the potential to allow widespread assessments of postural control in remote settings, with patients at home and/or at local clinics.

**Abstract:**

Background: There is a growing need for highly accessible and simplified methods to track postural control in adults affected by neurodegenerative diseases. Therefore, the aim of this study was to assess the validity of smartphone-derived postural control analyses compared with traditional center-of-pressure (COP) measures in healthy adults and people with type 2 diabetes mellitus (T2DM). Methods: A total of 36 participants (21 controls, 15 T2DM) completed static postural testing during single- and double-leg stance, also with eyes open and eyes closed. Data from a smartphone attached to the lower back measured trunk acceleration (SP-ACC) concurrently with gold-standard center of pressure (COP). The root mean square (RMS) and movement velocity (MV) were extracted from both trunk acceleration and COP data. The effect of balance condition and groups were statistically evaluated using non-parametric statistical tests. Results: SP-ACC and COP metrics showed progressive sway increases with task difficulty in both groups (all *p* < 0.001). RMS-ACC demonstrated moderate-to-strong correlations with RMS-COP across conditions (r = 0.55–0.90). Compared with controls, the T2DM group exhibited significantly higher RMS-ACC in DLS-EC and SLS-EO (both *p* < 0.01) and higher MV-ACC in DLS-EO, SLS-EO, and SLS-EC (*p* = 0.04–<0.001), reflecting impaired postural control. Conclusions: Smartphone-based IMU assessments showed good agreement with COP analysis and detected condition-specific balance deficits in T2DM. These findings support smartphone-based IMU metrics as a promising tool for accessible and scalable balance screening in diabetes care.

## 1. Introduction

Type 2 diabetes mellitus (T2DM) is a chronic metabolic disorder affecting more than 450 million adults globally. This figure is projected to grow, increasing from an estimated 6059 to 7079 cases per 100,000 individuals by 2030, with substantial economic burden in Europe alone [1,2]. T2DM is caused by insulin resistance and progressive β-cell dysfunction, with common sequelae such as peripheral neuropathy, pain, weakness, and compromised balance during daily activities [3,4]. Older adults with T2DM experience greater fall risk than their non-diabetic peers due to use of specific medications, cardiovascular disease, pain, reduced activity, cognitive limitations, and diminished lower-extremity performance [5,6,7]. Moreover, aging with T2DM can exacerbate visual and vestibular dysfunctions, as well as declines in peripheral sensory loss (including proprioceptive and cutaneous deficits). Therefore, T2DM drives deficits in neural mechanisms responsible to control posture, even in diabetics without clinically diagnosed peripheral neuropathy [8,9,10].

Motor strategies applied to recover balances such as ankle/hip strategies and anticipatory stepping are often compromised with age or disease [11]. Therefore, precise assessment of balance is central to fall prevention [12]. The most common assessments are inexpensive and practical clinical scales (e.g., the Berg Balance Scale, Timed Up and Go), which are limited by coarse scoring and reduced sensitivity to subtle impairments [13]. Objective posturography quantifies postural sway via center of pressure (COP) using ground reaction forces and moments, recorded using force plates [14,15,16]. Despite being considered gold-standard assessment tools, force plates are expensive, non-portable, complex to operate, and largely confined to lab settings, preventing widespread clinical use [15]. In between highly practical and highly expensive methods, there are objective yet affordable alternatives involving pressure platforms, vision-based systems, and inertial sensors [17,18,19]. Inertial measurement units (IMUs) are particularly promising for balance assessments, as a small IMU can measure linear and angular accelerations close to the body’s center of mass to retrieve postural sway. Previous studies have demonstrated good validity in using IMUs for assessments of postural sway [20] and measuring effects of visual deprivation in older adults [21].

IMU technology is present in contemporary smartphones, and balance assessment using the IMU embedded in smartphones has the potential to enable scalable, user-friendly and remote balance assessment [19]. Previous studies have demonstrated the feasibility and validity of using smartphone-based measures to detect postural alterations to prevent falls [22], postural stability in patients with multiple sclerosis [23], Parkinson’s disease [24], and stroke [25]. Regarding patients with T2DM, rigorous evaluation of smartphone-based sway metrics against gold-standard COP measures remains limited, despite their elevated fall risk. Therefore, research into validating smartphone-based postural sway analysis for this population is highly relevant.

The aim of this study was to determine the validity of smartphone-based static balance assessments in healthy adults as a control group (CONT) and patients with T2DM when compared to gold-standard COP-based balance assessment. More specifically, we investigated (i) whether smartphone-based analysis discriminates postural conditions in ways consistent with COP analysis, potentially highlighting CONT vs. T2DM group differences and (ii) whether smartphone- and COP-based data present agreement for both CONT and T2DM groups. We hypothesized that (1) smartphone-based balance assessment will accurately pinpoint deficits in balance control on both CONT and T2DM groups and that (2) the data from smartphone-based and COP-based analyses will be moderately/strongly associated, indicating agreement and validity between methods. Our results have the potential to advance practical fall risk assessment and continuous monitoring in T2DM care through inexpensive and scalable balance assessments using smartphones.

## 2. Materials and Methods

### 2.1. Participants

A total of 36 adults participated in this study. CONT consisted of 21 healthy adults (9 males, 12 females; mean age 23.3 ± 2.0 years; height 175.4 ± 10.0 cm; weight 71.5 ± 14.5 kg). The T2DM group comprised 15 participants (9 men, 6 women; mean age 72.3 ± 5.0 years; height 171.1 ± 9.3 cm; weight 93.7 ± 15.7 kg) enrolled through a local clinical diabetes center (Steno Diabetes Center North [SDCN], Denmark). Exclusion criteria for both groups were (1) recent history of musculoskeletal injuries in the lower limbs up to 6 months prior to the experiment and (2) inability to stand in two legs without support caused by vestibular dysfunctions. All participants received verbal and written instructions regarding the experimental procedures, and all participants provided informed consent. The study protocol was approved by the North Jutland Research department (F2024-197), North Denmark Region Committee on Health Research Ethics (N-20240025), and ClinicalTrials.gov (NCT06745544).

### 2.2. Experimental Procedure

Each participant performed four static balance tasks in a randomized sequence: double-leg stance with eyes open (DLS-EO), double-leg stance with eyes closed (DLS-EC), single-leg stance (dominant leg) with eyes open (SLS-EO), and single-leg stance with eyes closed (SLS-EC). Participants were instructed to attend the session using comfortable sports clothing/shoes. For each task, participants were instructed to maintain the requested posture for 30 s uninterruptedly. Prior to starting recordings, participants were instructed to focus on a target at their sight height placed on a wall ~5 m away, then assume the posture and stay as quiet as possible while maintaining their arms alongside the body. For eyes-closed conditions, participants were instructed to close their eyes when the experimenter indicated to start the recording. In case of loss of balance that required stepping to regain control during the recording, the trial was discarded, and a new trial was performed. For safety reasons, T2DM participants were permitted to perform the single-leg stance task with a horizontal support bar placed in front of them under both eyes-open and eyes-closed conditions. Participants were instructed only to lightly touch the bar and not fully support themselves using it during the trials. The use of the support bar was mandatory for all T2DM patients to minimize inconsistencies in the performance of their postural control tasks.

### 2.3. Instruments

The experimental recordings were performed in two different locations. The control group’s experiments were performed at a motion capture laboratory, and the COP data was acquired using its local force plate (BMS400600, AMTI, Watertown, MA, USA) sampled at 500 Hz. The T2DM group’s experiments were performed at SDCN, and the COP data was acquired using a portable force plate (Plux Biosignals, Lisbon, Portugal) sampled at 1000 Hz. Despite the different devices, both instruments have high accuracy and validity for the extraction of COP data; therefore, the comparison between groups was not compromised by the instrumentation. Regarding IMU gait assessments, the trunk acceleration from both controls and T2DM groups was acquired using a Samsung Galaxy A51 smartphone running a custom-made application for the extraction of the device’s IMU accelerometer data sampled at ≈63 Hz. The smartphone was fixed horizontally on the participants’ lower back (L5 level) using an elastic running belt to maximize comfort and standardize positioning.

### 2.4. Data Processing

The data from the smartphone’s IMU and the different force plates were analyzed using custom-made MATLAB scripts (MATLAB 2024b, The Mathworks, Natick, MA, USA). The COP data from the control group was extracted from the motion capture software (Oqus 300, 310, 500, Qualisys, Sweden) and low-pass-filtered within the software using a Butterworth filter (fourth-order, 10 Hz cut-off frequency). Similarly, forces and moments from the Biosignalplux force plate and the raw IMU accelerations were filtered using the same filter specifications using MATLAB. For the T2DM group, the COP data was calculated through scripts using previously established formulae [26]. The first and last 2.5 s of the COP and IMU recordings were excluded from the analysis to ensure stable posture across all recordings.

The acceleration data axes were realigned to correspond to vertical (X), mediolateral (Y), and anteroposterior (Z) directions. Subsequently, acceleration signals were detrended and the resultant acceleration (stated as √(|X| + |Y|)2) was calculated. The resultant accelerations were integrated using cumulative trapezoidal functions to generate velocities. This velocity signal was averaged throughout the 25-s data in each condition and divided by its duration to generate the average movement velocity (MV). In addition, the root mean square (RMS) values of the velocity signals were calculated to represent movement variability. Regarding COP data, a resultant COP signal was calculated using the X (mediolateral) and Y (anteroposterior) force data. Similarly to the IMU-based velocity analysis, the COP data was averaged across the 25-s data and divided by its duration in each condition to generate MV, and the same signal was used to calculate RMS values to represent its variability.

### 2.5. Statistical Analysis

Statistical analyses were performed in MATLAB R2024b. Normality was tested with the Shapiro–Wilk test. Since normality was not present across a substantial number of data samples, non-parametric statistics were applied, where the between-group and within-group effects were investigated separately. Firstly, the effects of conditions on MV-COP, MV-ACC, RMS-MV and RMS-ACC within controls and T2DM groups were assessed using the Friedman test. Differences between controls and T2DM groups were evaluated with Mann–Whitney U tests. Wilcoxon signed-rank tests with Bonferroni correction were used for post hoc analysis. Correlations between smartphone and force plate parameters were evaluated using Spearman’s or Pearson’s coefficients as appropriate and interpreted according to established thresholds [26].

## 3. Results

### 3.1. Effects of Postural Tasks on COP- and Smartphone-Based Variables

There was a consistent, progressive increase in sway as task challenge intensified for both CONT (Table 1) and T2DM groups (Table 2). Both RMS and MV values rose from DLS-EO to SLS-EC in both participant groups. For CONT, Friedman tests showed significant condition effects for all ACC and COP parameters (χ^2^ = 39.69–52.83, all *p* < 0.001). Post hoc Wilcoxon tests using Bonferroni correction confirmed significant differences between most condition pairs except for DLS-EO vs. DLS-EC (RMS-ACC *p* = 0.520), indicating the largest jumps in sway occurred with base-of-support reduction (SLS vs. DLS) and visual deprivation (EC vs. EO).

Among T2DM participants, condition effects were likewise significant for all parameters (χ^2^ = 17.4–36.1, all *p* < 0.001, Table 2). RMS and MV values increased most notably in single-leg conditions. Due to the need for physical support, data interpretation in these tasks (SLS-EO, SLS-EC) was limited. Significant post hoc differences were detected particularly between both-leg vs. single-leg tasks (RMS ACC, *p* < 0.001).

### 3.2. Agreement Between IMU- and COP-Based Variables

**Figure 1:**
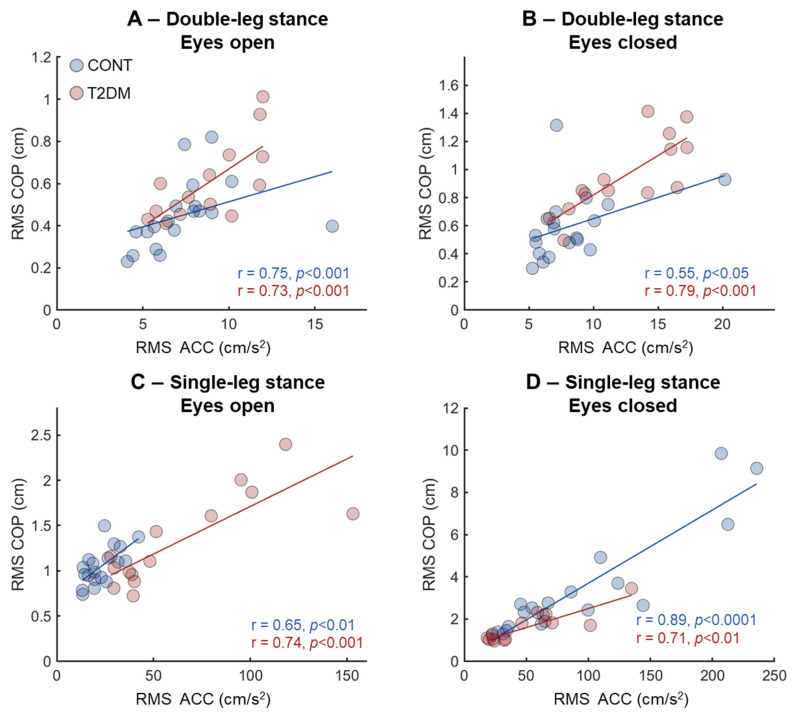
Pearson correlation coefficient from the associations between the root mean square extracted from the center of pressure (RMS COP) and extracted from the IMU smartphone accelerometer (RMS ACC) for the four different postural conditions (**A**–**D**). Associations were extracted from healthy participants (CONT, blue) and the type 2 diabetes mellitus group (T2DM, red).

In general, there were moderate-to-strong correlations across the different tasks for all groups when RMS COP and RMS ACC were compared. The DLS-EO condition showed similar associations for both groups (Figure 1A), whereas CONT presented only moderate correlation for DLS-EC (r = 0.55, Figure 1B). Associations during single-leg stance tasks were similar between groups with eyes open (Figure 1C), while CONT presented the highest association during SLS-EC (Figure 1D). The T2DM group presented strong correlations (r > 0.7) for all postural conditions investigated.

Regarding movement velocity (MV), there were generally weak associations for both groups between movement velocity calculated using COP data and trunk movement velocity calculated using smartphone IMU data for DLS-EO, DLS-EC and SLS-EO (Supplementary Table S1). As an exception, both groups presented significant moderate associations for SLS-EC (r > 0.55, *p* < 0.05).

### 3.3. Effect of T2DM on Postural Assessments

**Figure 2:**
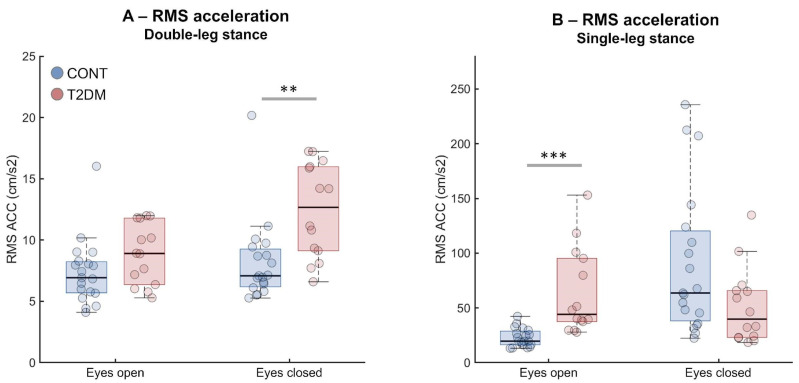
Boxplots representing root mean square from smartphone acceleration (RMS ACC) extracted from healthy participants (CONT, blue) and type 2 diabetes mellitus (T2DM, red) groups from double-leg stance (**A**) and single-leg stance tasks (**B**). Boxplots represent median, 25th and 75th percentile and data range (dashed vertical lines). Individual participants are represented by colored circles. Asterisks indicate statistically significant differences (** *p* < 0.01, *** *p* < 0.001).

Mann–Whitney U tests revealed significantly higher RMS ACC values in T2DM compared to CONT during DLS-EC (U = 48, r = 0.5, *p* = 0.002, Figure 2A) and SLS-EO (U = 18, r = 0.73, *p* < 0.001, Figure 2B). In the simplest task (DLS-EO), the difference was not statistically significant; in SLS-EC, CONT paradoxically showed higher RMS values, potentially reflecting the confounding influence of support required by T2DM subjects.

**Figure 3:**
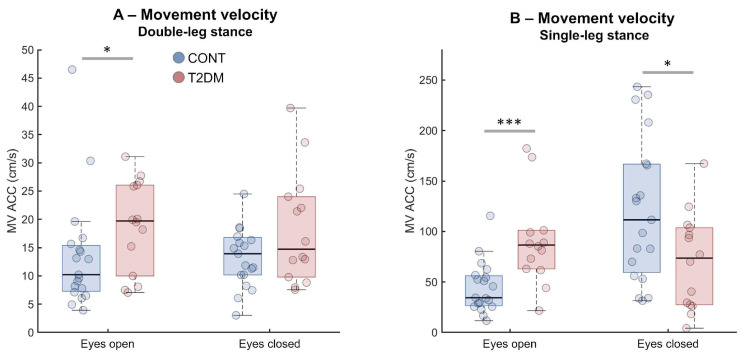
Boxplots representing average trunk movement velocity extracted using smartphone acceleration (MV ACC) from healthy (CONT, blue) and type 2 diabetes mellitus (T2DM, red) groups from double-leg stance (**A**) and single-leg stance tasks (**B**). Boxplots represent median, 25th and 75th percentile and data range (dashed vertical lines). Individual participants are represented by colored circles. Asterisks indicate statistically significant differences (* *p* < 0.05, *** *p* < 0.001).

Mann-Whitney U tests revealed significantly higher MV ACC values in the T2DM group compared to CONT during DLS-EO (U = 76, r = 0.36, *p* = 0.040, Figure 3A), SLS-EO (U = 41, r = 0.58, *p* = 0.001, Figure 3B), and SLS-EC (U = 74, r = 0.37, *p* = 0.033, Figure 3B). In these conditions, T2DM participants demonstrated greater trunk movement velocities, indicating increased postural instability. In DLS-EC, the difference in MV ACC values between groups was not statistically significant.

## 4. Discussion

Our study demonstrated that smartphone-based trunk acceleration metrics provide valid and sensitive estimates of static postural control in both healthy adults and people with T2DM when compared with gold-standard force plate COP analysis. RMS-ACC increased systematically with task difficulty and showed moderate-to-strong associations with RMS-COP across all conditions. Importantly, smartphone-based sway variability identified balance impairments in T2DM, particularly when visual input was removed or the base of support was reduced. Together, these findings indicate that the currently available smartphone sensors can detect clinically relevant balance deficits in T2DM by using simple data-processing algorithms, representing a practical screening solution for diabetes care.

### 4.1. Within-Group Differences Between Postural Tasks

Smartphone-based posturography demonstrated satisfactory condition sensitivity, tracking systematic increases in sway as tasks became more challenging (from DLS-EO to SLS-EC) and aligning with well-established visual contributions to balance. As vision was removed, both RMS and MV rose as expected for both COP-based and smartphone-based methods, consistent with prior evidence that visual input stabilizes upright stance [27,28,29]. On both healthy and T2DM groups, DLS-EO yielded the lowest sway magnitudes, whereas single-leg conditions—especially SLS-EC—elicited the greatest demands. An exception emerged for smartphone-based metrics in the T2DM cohort: peak RMS-ACC and MV-ACC occurred in SLS-EO rather than SLS-EC. This likely reflects participants’ reliance on physical support in SLS-EC, which reduced trunk excursions (captured by the IMU) even when plantar loading patterns (captured by the force plate) remained challenging. The divergence underscores a core measurement distinction: force plates quantify COP dynamics at the feet, whereas an IMU placed at the trunk captures its segmental motion; thus, strategy selection (ankle vs. hip) can differentially manifest in the two devices [30,31].

Mechanistically, ankle-strategy control tends to manifest as small COP shifts well detected on a force plate, whereas hip-strategy responses induce larger proximal motion more visible to a trunk-mounted IMU [32]. Aging and sensory degradation, including distal proprioceptive loss common in T2DM, can bias individuals toward greater reliance on hip strategies, particularly when the base-of-support narrows or surfaces destabilize [33,34]. Prior work in T2DM with sensory neuropathy showed medial–lateral–dominant sway components and increased COP displacement when vision was removed, consistent with a shift toward hip-dominant stabilization [35]. In the present study, the observed SLS patterns in T2DM are compatible with such strategy changes and with the moderating role of external support, which can mute trunk kinematics without equivalently reducing COP demands.

### 4.2. Agreement Between Smartphone IMU and Force Plate Metrics

There were moderate-to-strong associations between RMS-ACC and RMS-COP. The RMS is a widely used metric of signal variability, which has been previously used to assess static postural control in clinical populations [36]. Our results further demonstrate that RMS is a reliable descriptor of overall sway variability in healthy controls and patients with T2DM. In contrast, MV agreements strengthened primarily under the most demanding postural condition (SLS-EC), suggesting that velocity measures may be sensitive to rapid, larger postural corrections that emerge near balance limits [14,37]. Conversely, simpler postural tasks may demand milder postural corrections that elicit lower velocity fluctuations, subsequently eroding the agreement between ACC and COP metrics. Additionally, MV ACC required numerical integration of the acceleration signal, whereas MV COP arises from differentiation of COP trajectories. These are distinct operations with different noise propagation patterns that may help to explain weaker or condition-dependent agreements for MV [14,37,38].

### 4.3. Between-Group Differences in Postural Control

From a clinical–discriminative perspective, the smartphone-based analysis demonstrated expected deficits in T2DM: RMS and MV were generally greater in the T2DM group across most conditions, highlighting poorer postural control. In DLS-EO, between-group differences in RMS-ACC were small, consistent with the idea that redundancy among visual, vestibular, and somatosensory systems can preserve performance when at least two inputs remain intact [39]. However, removing visual input (DLS-EC) and narrowing base of support (SLS-EO) amplified group differences, aligning with the literature on sensory loss and increased sway in older and diabetic populations [40,41]. Our results alight with previous smartphone-based findings in T2DM showing higher RMS values relative to controls, with absolute value differences largely attributable to methodological choices (e.g., resultant vs. directional RMS; sensor placement) rather than contradictory physiology.

The only instance where CONT presented relatively poorer balance stability compared to T2DM occurred for RMS-ACC in SLS-EC (Figure 2B). This result is caused by the support aid provided for T2DM participants, which diminished their need to use trunk movements to maintain posture. Conversely, CONT participants produced larger hip-driven trunk swings due to the task difficulty level. MV patterns supported this interpretation: even when RMS differences were modest in simpler tasks, T2DM participants often showed greater MV-ACC, suggesting more frequent/abrupt corrective actions that raise velocity without necessarily inflating overall amplitude. In more challenging tasks, T2DM participants may have adopted slower, larger oscillations (support-buffered), driving RMS but not velocity to the same extent. Our results demonstrated that the force plate and smartphone consistently detected greater instability in T2DM under high-demand conditions, with the force plate sometimes differentiating groups even in DLS-EO owing to its sensitivity to subtle COP shifts [31].

Comparisons with other smartphone studies bolster the external validity of our approach. Our results corroborate previous studies demonstrating that healthy adults increase sway velocity with increased postural challenges (e.g., eyes closed, foam) for both smartphone and force plates [37]. Similarly, a study in at-risk older adults reported rising COP-MV and COM-related RMS with task difficulty [19]. Since T2DM is on the rise, regular, objective monitoring can identify at-risk individuals earlier, track disease-related declines, and quantify responsiveness to exercise and therapy that improve walking, reaction time, and balance [42,43]. Smartphones could embed this monitoring in everyday life and into telerehabilitation workflows, linking patients and clinicians, individualizing progression, and reducing unnecessary visits. Practically, smartphones can complement (not replace) force plates by providing scalable, low-cost monitoring that is sensitive to both task difficulty and pathology.

### 4.4. Clinical Relevance and Translation

Falls remain a significant concern in T2DM, with increased risk of injury, hospitalization, and functional decline. Early detection of subtle postural impairments is critical, yet conventional clinical scales often lack the sensitivity to capture these early changes. Our findings show that brief smartphone-based assessments can detect task-dependent balance deficits in T2DM and align well with validated COP measures. While larger studies are needed to establish predictive thresholds and clinical pathways, these findings indicate that smartphone-based IMU assessment may represent a feasible and accessible complement to existing balance evaluation approaches, particularly in settings where specialized posturography equipment is not available.

### 4.5. Limitations of the Study

The first relevant limitation in this study is the age difference between the control group (~23 years average) and the T2DM group (~72 years average). The postural control of the control group was superior when compared to the T2DM group, but the worsening of postural control in the T2DM group may also be caused by aging as a confounding factor. Nonetheless, the most relevant outcome from the study is the within-group validation analysis (e.g., condition’s sensitivity, COP-ACC agreements), which is unaffected by the age difference. Future studies assessing the sensitivity of smartphone-based postural control metrics should evaluate age-matched groups.

A second limitation of this study is the sample size, especially for the T2DM group. Their low sample size reflects the challenges in recruiting clinical populations. Future studies on the topic should target larger sample sizes to increase the power of the statistical analyses. Another limitation was that participants from the T2DM group were allowed to use a support bar to perform the single-leg standing tasks, whereas the control group did not use such support. The support bar was used as a safety measure, since the risk of a fall increased in the single-leg standing tasks, but it drastically modified the postural control strategy and undermined between-group comparisons. It would be expected that the T2DM group members would display the greatest RMS acceleration and movement velocities had they not used the support bar. Moreover, T2DM participants may have applied different pressures to the support bar while touching it, making the within-group comparisons for single-leg standing tasks inappropriate. Therefore, the only reliable between-group comparisons are from double-leg stance, where no support was provided. Future studies should standardize support conditions or use alternative safety measures. Moreover, having an instrumented bar that measures the force applied by the participant during testing would help to quantify the amount of assistance gained by touching/holding the bar. A third limitation is the lack of test–retest reliability, the solution of which will be the next step in our research.

## 5. Conclusions

In conclusion, this exploratory study demonstrated that smartphone-embedded IMU sensors were able to detect condition-dependent differences in static postural control in adults with T2DM and showed consistent alignment with gold-standard COP measures across standardized standing tasks. RMS-based sway metrics performed particularly well in reflecting postural variability. While these findings support the feasibility of smartphone-based assessments as an objective complement to existing balance evaluations, larger and more diverse studies remain necessary to confirm clinical utility, establish predictive thresholds, and determine optimal implementation in routine diabetes care.

## Figures and Tables

**Table 1:** Mean ± standard deviation (SD), and lower and upper bound of the 95% confidence interval (95% CI) from root mean square (RMS) and movement velocity (MV) values extracted from the smartphone accelerometer (ACC) and force plate’s center of pressure (COP) during the different balance conditions for the CONT group. † denotes significant difference in relation to SLS-EO (*p* < 0.01); * denotes significant difference in relation to SLS-EC (*p* < 0.01).

	Condition	Mean ± SD	95% CI
RMS-ACC (cm/s^2^)	DLS-EO	7.3 ± 2.7 † *	6.16–8.57
	DLS-EC	8.2 ± 3.3 † *	6.67–9.7
	SLS-EO	23.0 ± 8.3 *	19.22–26.73
	SLS-EC	90.0 ± 66.5	60.18–119.97
RMS-COP (cm)	DLS-EO	0.4 ± 0.1 † *	0.38–0.52
	DLS-EC	0.6 ± 0.2 † *	0.49–0.71
	SLS-EO	1.0 ± 0.2 *	0.96–1.14
	SLS-EC	3.3 ± 2.5	2.21–4.49
MV-ACC (cm/s)	DLS-EO	13.5 ± 10.1 † *	8.99–18.09
	DLS-EC	15.5 ± 12.1 † *	10.12–20.99
	SLS-EO	44.6 ± 26.0 *	33.27–55.98
	SLS-EC	121.2 ± 70.8	89.37–153.08
MV-COP (cm/s)	DLS-EO	1.0 ± 0.2 † *	0.87–1.11
	DLS-EC	1.3 ± 0.4 † *	1.12–1.54
	SLS-EO	4.3 ± 1.2 *	3.74–4.87
	SLS-EC	14.0 ± 9.2	9.88–18.19

**Table 2:** Mean ± standard deviation (SD), and lower and upper bound of the 95% confidence interval (95% CI) from root mean square (RMS) and movement velocity (MV) values extracted from the smartphone accelerometer (ACC) and force plate’s center of pressure (COP) during the different balance conditions for the T2DM group. ≠ denotes significant difference in relation to DLS-EC (*p* < 0.01); † denotes significant difference in relation to SLS-EO (*p* < 0.01); * denotes significant difference in relation to SLS-EC (*p* < 0.01).

	Condition	Mean ± SD	95% CI
RMS-ACC (cm/s^2^)	DLS-EO	8.8 ± 2.5 ≠ † *	8.9–10.15
	DLS-EC	12.4 ± 3.8 † *	12.67–14.45
	SLS-EO	63.5 ± 39.2	44.12–84.12
	SLS-EC	51.3 ± 34.5	39.79–69.33
RMS-COP (cm)	DLS-EO	0.6 ± 0.2 ≠ † *	0.56–0.70
	DLS-EC	0.9 ± 0.3	0.86–1.10
	SLS-EO	1.3 ± 0.5	1.13–1.59
	SLS-EC	1.6 ± 0.7	1.50–1.99
MV-ACC (cm/s)	DLS-EO	18.8 ± 8,2 †	19.72–23.07
	DLS-EC	18.2 ± 9.9 † *	14.72–23.43
	SLS-EO	107.9 ± 81.5	86.61-150.64
	SLS-EC	70.4 ± 47.6	73.65–95.31
MV-COP (cm/s)	DLS-EO	1.8 ± 0.6 ≠ † *	1.60–2.09
	DLS-EC	3.8 ± 1.8 † *	3.20–4.74
	SLS-EO	6.8 ± 3.7	4.83–8.72
	SLS-EC	7.4 ± 3.8	6.07–9.41

## Data Availability

Data will be available when requested.

## Supplementary Materials

The following supporting information can be downloaded at: https://www.mdpi.com/article/10.3390/s26092899/s1, Supplementary Table S1. Title: Pearson’s correlation coefficient (r) and significance level (p) from the association between body’s COP movement velocity calculated using ground reaction forces (Force plate COP), and trunk movement velocity calculated using smartphone’s IMU (Smartphone ACC) from the four different postural conditions. * denotes significant Pearson’s correlation.

## Abbreviations

The following abbreviations are used in this manuscript:

IMU	Inertial measurement unit
T2DM	Type 2 diabetes mellitus
CONT	Control group
GRF	Ground reaction force
OMC	Optical motion capture
COP	Center of pressure
ACC	Acceleration
DLS-EO	Double-leg standing–eyes open
DLS-EC	Double-leg standing–eyes closed
SLS-EO	Single-leg standing–eyes open
SLS-EC	Single-leg standing–eyes closed
RMS	Root mean square
MV	Mean velocity
