# Supplementary material for: An Exploratory Analysis of Postural Control in People with Type 2 Diabetes Mellitus Using a Smartphone IMU Sensor
Source: Sensors (Basel). 2026 May 6;26(9):2899. doi: 10.3390/s26092899 (PMC13166036; doi:10.3390/s26092899)
Supplement: Supplementary file 1 [file sensors-26-02899-s001.zip › sensors-4227748-supplementary.pdf]

Supplementary Table S1. Pearson's correlation coefficient (r) and significance level (p) from the association between body's COP movement velocity calculated using ground reaction forces (Force plate COP), and trunk movement velocity calculated using smartphone's IMU (Smartphone ACC) from the four different postural conditions. * denotes significant Pearson's correlation.

	**DLS-EO**	**DLS-EC**	**SLS-EO**	**SLS-EC**
**CONT**	r = 0.33, p = 0.16	r = 0.20, p = 0.40	r = 0.26, p = 0.26	r = 0.70, p<0.01*
**T2DM**	r = 0.28, p = 0.31	r = 0.06, p = 0.82	r = 0.41, p = 0.14	r = 0.55, p<0.05*
